# High glucose induces platelet‐derived growth factor‐C via carbohydrate response element‐binding protein in glomerular mesangial cells

**DOI:** 10.14814/phy2.12730

**Published:** 2016-03-31

**Authors:** Hiroya Kitsunai, Yuichi Makino, Hidemitsu Sakagami, Katsutoshi Mizumoto, Tsuyoshi Yanagimachi, Kuralay Atageldiyeva, Yasutaka Takeda, Yukihiro Fujita, Atsuko Abiko, Yumi Takiyama, Masakazu Haneda

**Affiliations:** ^1^ Division of Metabolism and Biosystemic ScienceDepartment of MedicineAsahikawa Medical UniversityAsahikawaJapan

**Keywords:** Carbohydrate response element‐binding protein, diabetic nephropathy, mesangial cells, platelet‐derived growth factor‐C, transcription factor

## Abstract

Persistent high concentration of glucose causes cellular stress and damage in diabetes via derangement of gene expressions. We previously reported high glucose activates hypoxia‐inducible factor‐1*α* and downstream gene expression in mesangial cells, leading to an extracellular matrix expansion in the glomeruli. A glucose‐responsive transcription factor carbohydrate response element‐binding protein (ChREBP) is a key mediator for such perturbation of gene regulation. To provide insight into glucose‐mediated gene regulation in mesangial cells, we performed chromatin immunoprecipitation followed by DNA microarray analysis and identified platelet‐derived growth factor‐C (PDGF‐C) as a novel target gene of ChREBP. In streptozotocin‐induced diabetic mice, glomerular cells showed a significant increase in PDGF‐C expression; the ratio of PDGF‐C‐positive cells to the total number glomerular cells demonstrated more than threefold increase when compared with control animals. In cultured human mesangial cells, high glucose enhanced expression of PDGF‐C protein by 1.9‐fold. Knock‐down of ChREBP abrogated this induction response. Upregulated PDGF‐C contributed to the production of type IV and type VI collagen, possibly via an autocrine mechanism. Interestingly, urinary PDGF‐C levels in diabetic model mice were significantly elevated in a fashion similar to urinary albumin. Taken together, we hypothesize that a high glucose‐mediated induction of PDGF‐C via ChREBP in mesangial cells contributes to the development of glomerular mesangial expansion in diabetes, which may provide a platform for novel predictive and therapeutic strategies for diabetic nephropathy.

## Introduction

Diabetic nephropathy (DN) is a major chronic microvascular complication of diabetes mellitus (DM) and is an important cause of increased morbidity and mortality in patients with DM. Previous studies including the Diabetes Control and Complications Trial in patients with type 1 diabetes (The Diabetes Control and Complications Trial Research Group 1993) and UK Prospective Diabetes Study in patients with type 2 diabetes (Turner 1998) indicate a causal link between the degree of glycemic control in patients with diabetes and the development and progression of DN. In addition, Action to Control Cardiovascular Risk in Diabetes, a recent large intervention trial aiming at tight glycemic control, demonstrated a reduction in the risk of new‐onset albuminuria and surrogate outcomes of DN (Ismail‐Beigi et al. 2010). Obviously, a remedy for hyperglycemia could prevent the initiation and development of DN. Elucidation of the hyperglycemia‐related molecular pathogenesis of organ damage would provide further insight into therapeutic strategies for DN.

The histopathologic hallmarks of DN are increased thickness of the glomerular basement membrane and an excessive accumulation of extracellular matrix proteins in the glomeruli (Mason and Wahab 2003). Multiple mechanisms including hyperglycemia‐induced metabolic and hemodynamic changes (Gnudi et al. 2003) and genetic predisposition (Mueller et al. 2006) have been shown to induce functional changes in glomerular mesangial cells. High glucose is a primary initiating factor of hyperactivation of metabolic pathways for polyols (Dunlop 2000) and hexosamines (Schleicher and Weigert 2000), the generation of advanced glycation end products (Forbes et al. 2003), and reactive oxygen species (Kiritoshi et al. 2003). In addition, high glucose evokes an activation of protein kinase C (Haneda et al. 2001), production of cytokines, such as transforming growth factor‐*β*1 (TGF‐*β*1) and connective tissue growth factor (CTGF) (Murphy et al. 1999), leading to the overproduction of matrix proteins by mesangial cells (Haneda et al. 2003). High glucose induces inhibitors of matrix‐degrading enzymes including plasminogen activator inhibitor‐1 (PAI‐1), which also leads to an accumulation of extracellular matrix proteins (Lee and Ha 2005).

Recently, several factors including SMAD1, SMAD3, STAT1, STAT3, Nrf2, and NF*κ*B have been shown to participate in gene regulation in the kidneys of diabetic patients or diabetic animal models (Hong et al. 2001; Mima et al. 2008; Yang et al. 2008; Berthier et al. 2009; Cui et al. 2012). Such transcriptional regulators are possibly downstream of hyperglycemia and thus may emerge as attractive targets in therapeutic attempts. In line with this, we previously demonstrated an activation of the hypoxia‐inducible transcription factor‐1*α* (HIF‐1*α*) by high glucose without the aid of hypoxia in glomerular mesangial cells (Isoe et al. 2010). In cultured mesangial cells, high glucose enhanced the expression of HIF‐1*α* and genes, such as CTGF and PAI‐1, which are known to be involved in extracellular matrix accumulation in diabetic glomeruli, indicating a previously unknown role of HIF‐1*α* in the development of glomerulopathy in response to high glucose. Of note, a glucose‐responsive carbohydrate response element‐binding protein (ChREBP) was found to upregulate HIF‐1*α* mRNA expression via direct binding to the promoter region of the HIF‐1*α* gene, providing a mechanism for diverse output of glucose signaling and a novel link between high glucose and diabetic kidney injury.

ChREBP is a basic helix‐loop‐helix/leucine zipper transcription factor. ChREBP is expressed in several metabolically relevant tissues, including adipocytes, pancreatic *β*‐cells, and certain cancer cells, indicating a wide variety of roles and its physiological importance (He et al. 2004; Noordeen et al. 2010; Wu et al. 2013). Upon activation by glucose, ChREBP translocates from the cytosol into the nucleus (Kawaguchi et al. 2001). In the nucleus, ChREBP forms a heterodimer with Max‐like protein X (Mlx) to bind to the carbohydrate response element (ChRE) for transcriptional regulation of its target genes (Shih et al. 1995; Stoeckman et al. 2004; Ma et al. 2006). Jeong et al. (2011) demonstrated ChRE motif search and identified 1153 ChREBP‐binding sites and 783 target genes using the chromatin from HepG2, a human hepatocellular carcinoma cell line. Gene ontology analysis showed that ChREBP target genes in hepatocytes are particularly associated with lipids, fatty acid, and steroid metabolism. Genes regulated in mesangial cells via ChREBP, however, have not been documented.

To provide more insight into glucose‐responsive gene regulation in mesangial cells, we performed a genome‐wide search for ChREBP‐binding sites employing chromatin from human mesangial cells exposed to high glucose and demonstrated the possible role of ChREBP in the profibrotic response of mesangial cells to high glucose ambience.

## Materials and Methods

### Reagents and cells

Human mesangial cells were obtained from Lonza (Basel, Switzerland). The immortalized mouse mesangial cells, SV40 MES 13, were from the American Type Culture Collection (Manassas, VA). Recombinant human PDGF‐C was obtained from PeproTech (Rocky Hill, NJ). 6,7‐Dimethyl‐2‐phenylquinoxaline (AG1295) was purchased from Calbiochem (Darmstadt, Germany).

### Cell culture

Before each assay, the cells were serum starved for 12 h in media containing 0.2% bovine serum albumin. Cellular responses to different glucose concentrations were determined in the serum‐free culture media.

### Chromatin immunoprecipitation and microarray

Human mesangial cells cultured in 5.6 or 25 mmol/L glucose were cross‐linked with formaldehyde. Cross‐linking was stopped by the addition of glycine. Nuclei were collected and resuspended in a sonication buffer, and sonicated using Bioruptor UCD‐250 (COSMO BIO, Tokyo, Japan). After sonication, the chromatin solution was incubated with Dynabeads protein G and rabbit anti‐ChREBP (Novus biologicals, Littleton, CO) or rabbit normal IgG at 4°C overnight. Antibody‐bound complexes were obtained, and DNA fragments extricated from these complexes were purified using a ChIP‐IT Express Kit (Active Motif, Carlsbad, CA). The purified immunoprecipitated DNA samples were analyzed by microarray hybridization (ChIP‐chip) assays using an Affymetrix GeneChip Human 2.0R Array Set (Santa Clara, CA). PCR analysis was performed using primers flanking the ChRE within the PDGF‐C gene promoter: human, 5′‐TAGTCTGAAATGAAACACACA‐3′ (forward) and 5′‐ACTTTTCTGACTCTCTGCTTG‐3′ (reverse), mouse, 5′‐CCAACTACAAGATGCACACA‐3′ (forward) and, 5′‐CAATAGTGAGATCGAGACTG‐3′ (reverse).

### Quantitative PCR analyses

Total RNA was reverse transcribed into cDNA with oligo‐dT primers using Superscript II reverse transcriptase (Invitrogen, Carlsbad, CA). Quantitative PCR was performed using *β*‐actin as an internal standard on the basis of TaqMan Gene Expression Assays (Applied Biosystems, Foster City, CA).

### Immunobloting

The proteins extracted from whole cells were separated on sodium dodecyl sulfate‐polyacrylamide gel electrophoresis and transferred to a polyvinylidene difluoride membrane. For western blots, the following primary antibodies were used: anti‐PDGF‐C (Abcam, Cambridge, UK), anti‐Collagen IV (Abcam), anti‐Collagen VI (Abcam), and anti‐b‐actin (Santa Cruz, Dallas, TX) and they were diluted 1:1000. Primary antibodies were incubated in Tris‐buffered saline with 1% milk. Secondary antibodies were conjugated with horseradish peroxidase (GE Healthcare, Buckinghamshire, UK), diluted 1:20000, and an electrochemiluminescent detection system (GE Healthcare) was used for visualization. For the semiquantitative analyses, the band densities were measured using Multigauge ver. 2.2 (Fujifilm, Tokyo, Japan).

### Gene knockdown by shRNA

To knockdown ChREBP or PDGF‐C, mouse mesangial cells were transfected with small hairpin RNA (shRNA) specifically targeting ChREBP or PDGF‐C. The sequences of the RNA are as follows: ChREBP sense, 5′‐GGCCUCAAGUUGCUAUGCCTT‐3′ and antisense, 5′‐GGCAUAGCAACUUGAGGCCTT‐3′; PDGF‐C sense, 5′‐GAAATATGGTGCTGGTGTG‐3′ and antisense, 5′‐CACACCAGCACCATATTTCTT‐3′. shRNA with a nontargeting scrambled sequence used as a control.

### Animals

Male C57BL/6 mice, 6 weeks old, weighing 20–22 g were rendered diabetic by the intraperitoneal injection of 50 mg streptozotocin (STZ) per kg body weight in a citrate buffer, pH 4.5, for 5 consecutive days. The diabetic state was confirmed 2 weeks after final injection by the measurement of their blood glucose level. All mice that were given STZ had a blood glucose concentration exceeding 300 mg/dL and were considered diabetic. Genetically diabetic male BKS.Cg‐+Leprdb/+Leprdb/J (db/db) mice and their age‐matched heterozygous non‐diabetic male littermates BKS.Cg‐Dock7m+/+Leprdb/J (db/m) were obtained from Charles River Laboratories Japan (Yokohama, Japan). All animal experiments were conducted according to the institutional guidelines for animal care and welfare.

### Histological analyses of the kidney

The kidneys of the mice were fixed in 4% paraformaldehyde, dehydrated, and embedded in paraffin. Then, 4 *μ*m sections were used for Periodic acid‐Schiff (PAS) staining and immunohistochemical analysis. Immunohistochemical analyses with polyclonal anti‐PDGF‐C antibody (R & D systems, Minneapolis, MN) were carried out as described (Makino et al. 2003). For semiquantitative analyses, 30 glomeruli were used to count PDGF‐C‐positive cells to determine the number of positive cells per glomerulus and the ratio of positive cells to the total number of cells. Immunofluorescent analyses were performed by incubation with polyclonal anti‐PDGF‐C antibody (Santa Cruz), anti–*α*‐smooth muscle actin (*α*‐SMA) antibody (Abcam), monoclonal anti‐CD34 antibody (Abcam), or polyclonal anti‐nephrin antibody (PROGEN Biotechnik GmbH, Heidelberg, Germany) followed by fluorescence‐conjugated secondary antibodies. Staining with 4,6‐diamidino‐2‐phenylindole (DAPI) was performed to identify the nucleus.

### Enzyme‐linked immunosorbent assay

Spot urine samples and random serum samples were obtained from the mice. Urinary PDGF‐C levels and plasma PDGF‐C levels were measured using an ELISA kit for platelet derived growth factor‐C (PDGF‐C) (Uscn Life Science, Wuhan, China). Urinary albumin levels were measured using Albumin Mouse Urine ELISA kit, Albuwell M (Exocell, Philadelphia, PA). The values of their urinary PDGF‐C levels and albumin levels were expressed as values corrected by the urinary creatinine concentration measured by a Creatinine Companion Kit (Exocell).

### Statistical analysis

All data are presented as means ± SD from the experiments with repetitions as indicated. Comparisons between two groups were analyzed using a *t*‐test. One‐factor analysis of variance and two‐factor factorial analysis of variance followed by the Bonferroni/Dunn test were carried out to determine significant differences in multiple comparisons. A statistical *P*‐values of <0.05 were considered to be significant.

## Results

### Genome‐wide chromatin immunoprecipitation analysis identified PDGF‐C as a target gene of ChREBP in glomerular mesangial cells in high glucose ambience

To explore the role of ChREBP in transcriptional regulation in glomerular mesangial cells in diabetes, we performed a genome location and expression profiling analysis to identify genes directly regulated by ChREBP. We treated primary human mesangial cells with high levels of glucose (25 mmol/L) for 48 h, then performed three independent chromatin immunoprecipitations (ChIP) with anti‐ChREBP antibodies followed by microarray hybridization assays (ChIP‐chip) to assess the extent of promoter occupancy by ChREBP at a genome‐wide level. As a control, we carried out parallel ChIP‐chip experiments employing mesangial cells treated with a media containing normal levels of glucose (5.6 mmol/L). Among a total of 87 genes selectively enriched with anti‐ChREBP antibodies in mesangial cells cultured under high glucose conditions (Table S1), PDGF‐C gene was found to harbor ChREBP‐binding sites within 5 kbp of the 3′‐flanking region. Accordingly, sequence analysis of human *PDGF‐C* gene revealed the presence of a ChRE‐like sequence at approximately 3.0 kbp downstream of the PDGF‐C gene.

**Figure 1:**
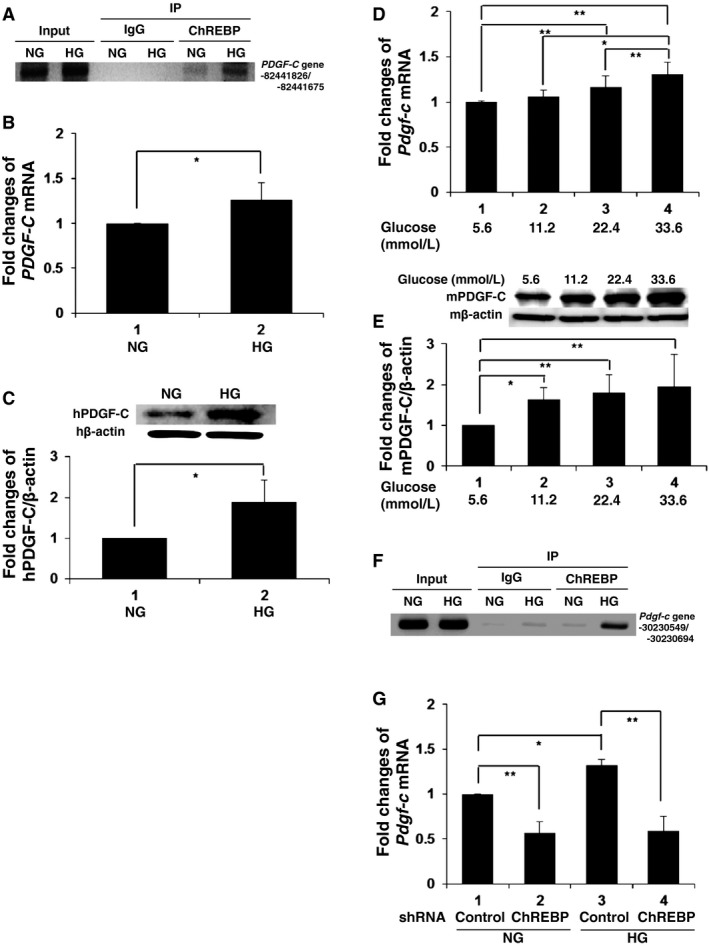
High glucose induces expression of platelet‐derived growth factor‐C (PDGF‐C) via ChREBP in glomerular mesangial cells. (A, F) Chromatin immunoprecipitation (ChIP) assays. Human mesangial cells (hMC) (A) or mouse mesangial cells (mMC) (F) were cultured in either normal glucose medium (NG; 5.6 mmol/L) or high glucose medium (HG; 25 mmol/L) for 48 h. ChIP assays using anti‐ChREBP antibody were then performed and rabbit polyclonal IgG (IgG) was applied as a control. PCR products spanning the indicated region of the PDGF‐C gene promoter for 40 cycles were separated by electrophoresis. (B, C) hMC were incubated in NG or HG medium for 48 h. mRNA expression of human *PDGF‐C* was determined by real‐time PCR. The means ± SD of mRNA levels relative to cells in NG medium are presented. *P < 0.05; *n* = 4 (B). Immunoblot analyses for expression of human PDGF‐C (hPDGF‐C) and the mean ± SD of the relative expression level of hPDGF‐C to *β*‐actin level. *P < 0.05; *n* = 4 (C). (D, E) mMC were incubated in the medium containing the indicated concentrations of glucose for 24 h. (D) mRNA expression of mouse *Pdgf‐c* was determined by real‐time PCR. The means ± SD of mRNA levels relative to cells in NG medium are presented. *P < 0.05; **P < 0.01; *n* = 6. (E) Immunoblot analyses for expression of mouse PDGF‐C (mPDGF‐C) and the mean ± SD of the relative expression level of mPDGF‐C to *β*‐actin level. *P = 0.05; **P < 0.05; *n* = 4. (G) mMC transfected with small ChREBP hairpin RNA (shRNA) expression vector were incubated for 24 h in either NG or HG medium and the *Pdgf‐c* mRNA levels were determined by real‐time PCR. shRNA with nontargeting sequence was used as a control. The means ± SD of the mRNA levels relative to cells in control shRNA‐transfected cells in the NG medium are presented. *P < 0.05; **P < 0.01; *n* = 4.

Given these facts, we performed conventional ChIP analyses and demonstrated ChREBP binding to this sequence in human mesangial cells cultured in high glucose media (Fig. 1A). To validate the results of the ChIP‐chip assay, we determined PDGF‐C expression in human mesangial cells in response to high‐glucose stimulation. Quantitative PCR demonstrated 1.3‐fold induction of *PDGF‐C* mRNA in human measangial cells cultured in high glucose media compared to the cells in normal glucose media (Fig. 1B). Similarly, immunoblot analyses showed a 1.9‐fold increase in PDGF‐C protein levels in response to high glucose media compared to normal glucose (Fig. 1C). Analogous to human cells, mouse mesangial cells showed an induction response to *Pdgf‐c* mRNA upon stimulation with glucose in a concentration‐dependent manner (Fig. 1D). Consistently, protein levels of PDGF‐C were dose dependently upregulated by glucose; 11.2 mmol/L and higher concentrations of glucose gradually induced a significant increase in PDGF‐C expression (Fig. 1E). In support of these observations, sequence analyses found the ChRE‐like sequence in the first intron of mouse *Pdgf‐c* genes and ChIP assays adopting mouse mesangial cells. This demonstrated high glucose‐dependent binding of ChREBP to the site (Fig. 1F). Moreover, the shRNA‐mediated reduction in cellular ChREBP levels in mouse mesangial cells resulted in an impairment of basal and high glucose‐induced *Pdgf‐c* mRNA expression (Fig. 1G). These results indicate that high glucose upregulates PDGF‐C expression in glomerular mesangial cells via direct regulation by ChREBP.

### PDGF‐C expression is elevated in glomerular mesangial cells of diabetic model mice

**Figure 2:**
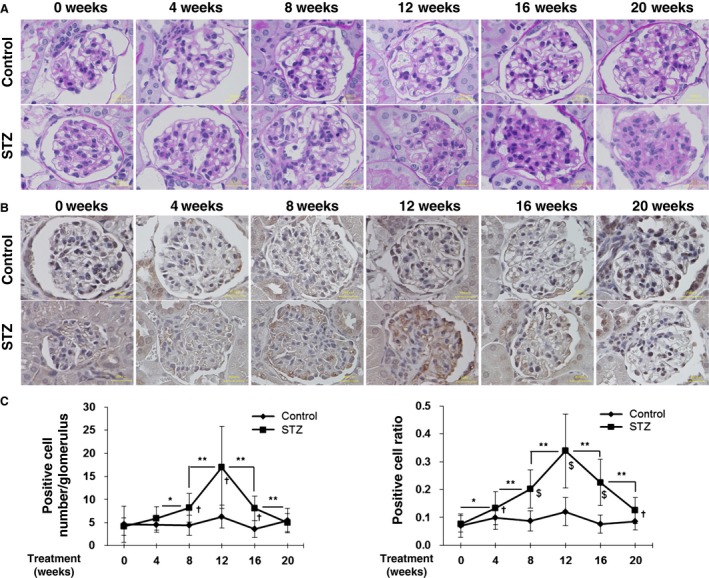
Platelet‐derived growth factor‐C (PDGF‐C) is expressed in the glomeruli of diabetic model mice. (A, B) Streptozotocin (STZ)‐induced diabetic and control mice were dissected at 0–20 weeks after treatment as indicated, and Periodic acid‐Schiff staining (A) and immunohistochemical analyses for PDGF‐C expression (B) in the glomeruli were performed. Bars = 20 *μ*m. (C) Semiquantitative analyses for PDGF‐C positive cells were performed by analyzing 30 glomeruli. Left panel; the mean ± SD number of the positive cells per glomerulus is presented. *P < 0.05; **P < 0.01; †*P* < 0.01 (STZ vs. control). Right panel; the ratio of positive cells to the total number of counted cells is indicated. *P < 0.05; **P < 0.01; †*P* < 0.05 (STZ vs. control); $*P* < 0.01 (STZ vs. control).

**Figure 3:**
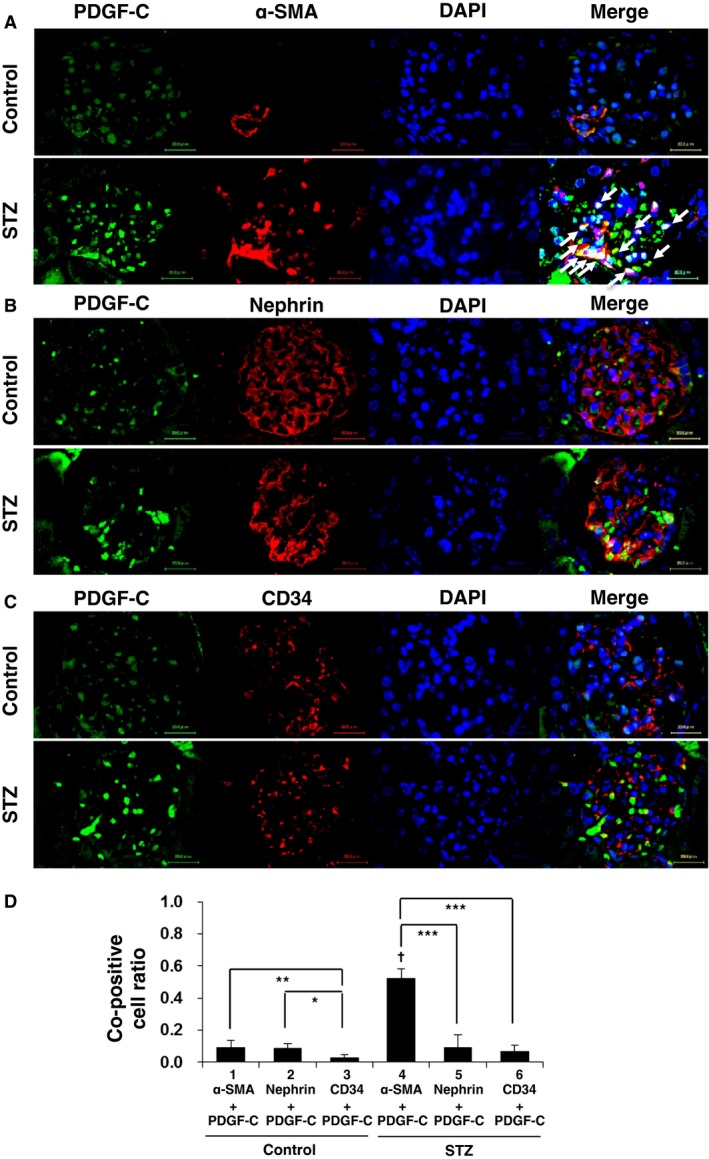
Platelet‐derived growth factor‐C (PDGF‐C) is expressed in mesangial cells in the glomeruli of diabetic model mice. (A–C) Immunofluorescent study for PDGF‐C, *α*‐smooth muscle actin (*α*‐SMA) (A), nephrin (B), and CD34 (C) expression in the glomeruli of streptozotocin (STZ)‐induced diabetic mice and control mice at 12 weeks after STZ treatment. Bars = 20 *μ*m. 4, 6‐diamidino‐2‐phenylindole (DAPI) signals depict localization of the nucleus. (D) Semiquantitative analyses of the cells copositive for PDGF‐C and for each marker were performed by analyzing 10 glomeruli to determine the ratio of copositive cells to the total number of the counted cells. *P < 0.1; **P < 0.05; ***P < 0.01; †*P* < 0.01 (STZ vs. control).

**Figure 4:**
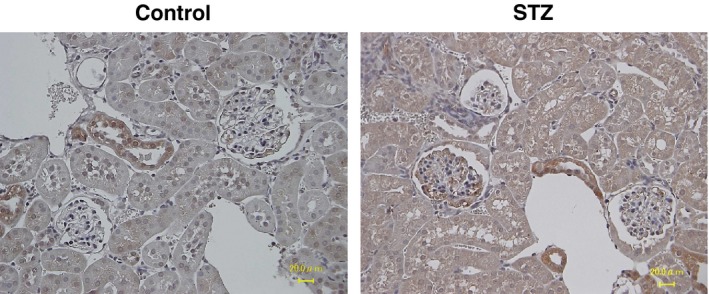
Platelet‐derived growth factor‐C (PDGF‐C) expression in tubulointerstital area. Immunohistochemical analyses for PDGF‐C expression in the kidney of streptozotocin (STZ)‐induced diabetic mice and control mice were performed. Bars = 20 *μ*m.

Next we examined the histological change and the expression of PDGF‐C in the glomeruli of diabetic model mice. Kidneys from diabetic and non‐diabetic mice after STZ or vehicle injection were analyzed by PAS staining. At 8 weeks after treatment, an increase in the glomerular cellularity became evident. At 12 weeks and later, expansions of the extracellular matrix in the glomeruli were observed (Fig. 2A). On the other hand, at 4 weeks after treatment, a modest staining of PDGF‐C was observed in the glomeruli of both STZ‐ and vehicle‐treated mice (Fig. 2B). In the glomeruli of mice at 8, 12, and 16 weeks after STZ introduction, PDGF‐C signals were higher than that in the glomeruli of the control mice. At 20 weeks after the STZ injections, PDGF‐C staining was scarcely detectable, as it was in the control mice at the same time point. In a series of semiquantitative analyses, PDGF‐C positive cells were found with a significantly higher incidence in diabetic kidneys compared to the control mice between 8 and 16 weeks after the STZ injections. The number of PDGF‐C positive cells was highest at 12 weeks after the introduction of STZ (Fig. 2C). The ratio of PDGF‐C‐positive cells to the total number glomerular cells was significantly increased in the same time course in diabetic mice (Fig. 2C); therefore cellular hyperplasia does not explain the increase in the number of PDGF‐C positive cells. To characterize the cells expressing PDGF‐C in the glomeruli, we performed coimmunofluorescent staining. The number of the cells with positive staining for PDGF‐C or *α*‐SMA, which is often induced in mesangial cells in injured glomeruli, was increased in STZ‐induced diabetic mice (Fig. 3A). When the fluoromicroscopic photographs were merged, a certain fraction of PDGF‐C staining colocalized with *α*‐SMA staining (Fig. 3A, arrows), indicating an increase in PDGF‐C in the mesangial cells in diabetic kidneys. Costaining of PDGF‐C and nephrin, a marker of podocytes, depicted only a few overlaps of PDGF‐C and nephrin signals (Fig. 3B). The costaining with CD34, a marker of vascular endothelial cells, show a limited presence of induced PDGF‐C in endothelial cells in the glomeruli (Fig. 3C). A semiquantitative summary of these coimmunofluorescent studies revealed that PDGF‐C is most abundantly expressed in mesangial cells in diabetic glomeruli (Fig. 3D). These results suggest that PDGF‐C may participate in the pathology of glomerular mesangial cells in diabetic kidneys. On the other hand, in the tubulointerstitial area, PDGF‐C expression was observed only in distal tubules. The extent of PDGF‐C staining was the same in diabetic and non‐diabetic mice throughout the time course after animal model induction (Fig. 4), indicating that PDGF‐C is constitutively expressed in the distal tubules.

### High glucose‐induced PDGF‐C upregulates genes encoding Collagen IV and Collagen VI in mesangial cells

**Figure 5:**
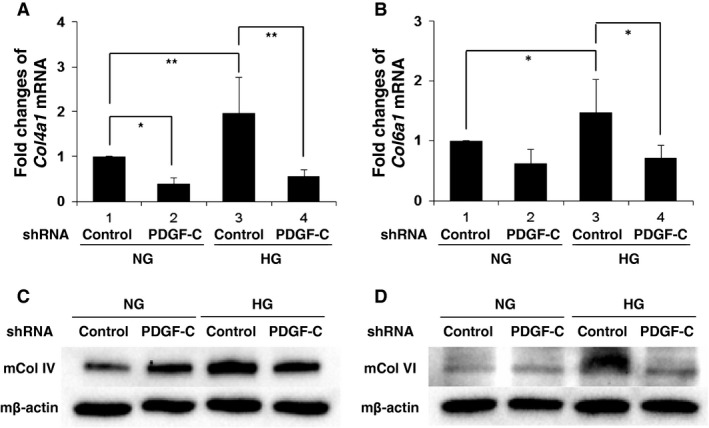
Platelet‐derived growth factor‐C (PDGF‐C) knockdown reduces high glucose‐induced upregulation of type IV collagen and type VI collagen expression in mesangial cells. (A–D) Small hairpin RNA (shRNA) targeting PDGF‐C was introduced to mouse mesangial cells, and then the cells were incubated in normal glucose medium (NG; 5.6 mmol/L) or high glucose medium (HG; 25 mmol/L) for 24 h. A nontarget shRNA was used as a control. (A) Mouse *Col4a1* (*Col4a1*) mRNA levels were determined by real‐time PCR. The means ± SD of the mRNA levels relative to cells in control shRNA‐transfected cells in an NG medium are presented. *P < 0.05; **P < 0.01; *n* = 5. (B) Mouse *Col6a1* (*Col6a1*) mRNA levels were determined by real‐time PCR. The means ± SD of the mRNA levels relative to cells in control shRNA‐transfected cells in NG medium are presented. *P < 0.05; *n* = 5. (C) Mouse type IV collagen (mCol IV) and (D) mouse type VI collagen (mCol VI) expression were determined by immnoblot.

Recent studies have shown that PDGF‐C has critical roles in mitogenic and fibrogenic changes in various disease conditions including lung fibrosis or liver cirrhosis. In this context, PDGF‐C is known to participate in the regulation of genes‐encoding type IV or type VI collagens. Therefore, we investigated the role of high glucose‐induced PDGF‐C in mesangial cells with respect to an accumulation of extracellular matrix or fibrotic components. For this purpose, we generated mouse mesangial cells in which PDGF‐C expression is compromised by the stable integration of a PDGF‐C shRNA expression vector. Culture of mouse mesangial cells in a medium containing a high concentration (25 mmol/L) of glucose induces higher expression of mouse *Col4a1* mRNA or *Col6a1* mRNA, compared to cells in a medium with a normal concentration (5.6 mmol/L) of glucose (Fig. 5A; lane 3 compared to lane 1, Fig. 5B; lane 3 compared to lane 1, respectively). Knockdown of PDGF‐C abrogated both basal and high glucose‐induced expression of mouse *Col4a1* mRNA and *Col6a1* mRNA (Fig. 5A; lanes 4 and 2 compared to lanes 3 and 1, Fig. 5B; lanes 4 and 2 compared to lanes 3 and 1, respectively). Similarly, mouse‐type IV collagen (mCol IV) and type VI collagen (mCol VI) proteins were upregulated in mesangial cells exposed to high glucose (Fig. 5C; lane 3 compared to lane 1, Fig. 5D; lane 3 compared to lane 1, respectively) and reduction in cellular PDGF‐C impaired such cellular induction response to high glucose (Fig. 5C; lane 4 compared to lane 3, Fig. 5D; lane 4 compared to lane 3, respectively).

**Figure 6:**
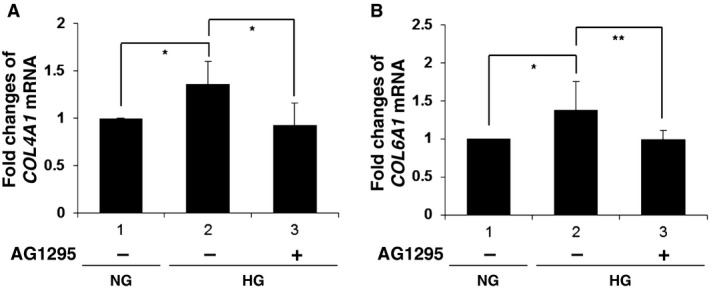
Platelet‐derived growth factor receptor inhibitor reduces high glucose‐induced upregulation of *Col4a‐1* and *Col6a‐1* gene expression in mesangial cells. (A, B) Cultured human mesangial cells were incubated in normal glucose medium (NG; 5.6 mmol/L) or high glucose medium (HG; 25 mmol/L) in the presence (+) or absence (−) of 10 *μ*mol/L 6, 7‐Dimethyl‐2‐phenylquinoxaline (AG1295) for 24 h. (A) Real‐time PCR analysis of *COL4A1* mRNA expression was performed. The means ± SD of the mRNA levels relative to cells in NG medium are presented. *P < 0.01; *n* = 7. (B) Real‐time PCR analysis of *COL6A1* mRNA expression was performed. The means ± SD of the mRNA levels relative to cells in NG medium are presented. *P < 0.05; **P < 0.01; *n* = 7.

**Figure 7:**
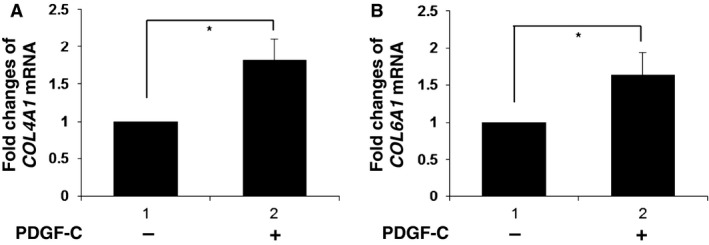
Platelet‐derived growth factor‐C (PDGF‐C)‐mediated regulation of type IV collagen or type VI collagen expression in mesangial cells. (A, B) Cultured human mesangial cells were incubated in normal glucose medium (NG; 5.6 mmol/L) in the presence (+) or absence (−) of 40 ng/mL PDGF‐C for 24 h. (A) Real‐time PCR analysis of *COL4A1* mRNA expression was performed. The mean ± SD of the mRNA levels relative to cells in the absence of PDGF‐C is presented. *P < 0.01; *n* = 5. (B) Real‐time PCR analysis of *COL6A1* mRNA expression was performed. The mean ± SD of the mRNA levels relative to cells in the absence of PDGF‐C is presented. *P < 0.01; *n* = 5.

These results indicate the critical role of PDGF‐C in a high glucose‐mediated induction of type IV collagen and type VI collagen in glomerular mesangial cells. In accord with this hypothesis, the addition to the culture medium of an inhibitor of PDGF‐*α* and ‐*β* receptors, AG1295, abolished high glucose‐induced upregulation of *COL4A1* and *COL6A1* mRNA expression in human mesangial cells (Fig. 6A and B, compared lane 3 to lane 2). Moreover, stimulation of human mesangial cells with PDGF‐C caused an induction of *COL4A1* and *COL6A1* mRNA (Fig. 7A and B). Taken together, PDGF‐C and the cognate receptor‐mediated signals are crucially involved in an augmentation of extracellular matrix protein production by mesangial cells in high glucose ambience.

### Urinary PDGF‐C levels are increased in diabetic model mice

**Figure 8:**
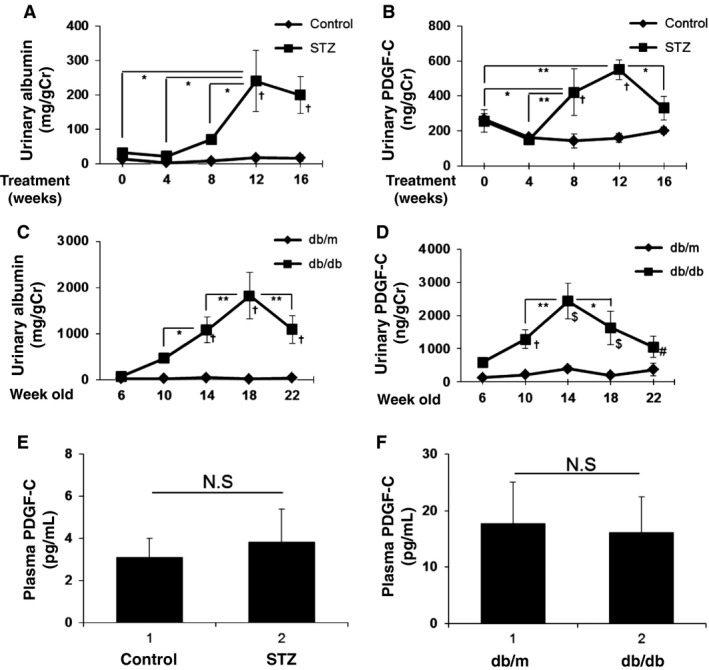
Urinary platelet‐derived growth factor‐C (PDGF‐C) level is increased in diabetic model mice. (A, B) Urinary albumin (A) or PDGF‐C (B) levels of streptozotocin (STZ)‐induced diabetic mice and control mice were measured by ELISA at 0–16 weeks after treatment as indicated. Results are expressed as an albumin–creatinine ratio (mg/gCr) and PDGF‐C–creatinine ratio (ng/gCr). *P < 0.01; †*P* < 0.01 (STZ vs. control); *n* = 6–14 (A) and *P < 0.05; **P < 0.01; **†** *P* < 0.01 (STZ vs. control); *n* = 6–14 (B). (C, D) Urinary albumin (C) or PDGF‐C (D) levels of db/db mice and db/m mice were measured at 6–22 weeks after birth. *P < 0.1; **P < 0.05; †*P* < 0.01 (db/db vs. db/m); *n* = 8–12 (C) and *P < 0.1; **P < 0.05; †*P* < 0.05; $*P* < 0.01; #*P* < 0.1 (db/db vs. db/m); *n* = 8–12 (D). (E, F) Plasma PDGF‐C levels of STZ‐induced diabetic mice and control mice (12 weeks after treatment) (E) or db/db mice and db/m mice (14‐week‐old) (F) were measured. *n* = 4.

Finally, we determined levels of urinary PDGF‐C in diabetic model mice. In type 1 diabetes model mice induced by STZ treatment, urinary albumin excretion was detectable at 8 weeks after treatment, then significantly increased compared to the control group at 12 weeks or later, indicating the possible presence of kidney damage in the model mice (Fig. 8A). Urinary PDGF‐C levels were significantly elevated at 8 weeks and reached a peak at 12 weeks after induction (Fig. 8B), showing a similar or earlier profile of urinary appearance compared to albumin. In contrast, plasma PDGF‐C levels were not elevated even at 12 weeks after STZ treatment (Fig. 8E), indicating that the increase in urinary PDGF‐C may reflect local events in the diabetic kidney. Type 2 diabetes model mice, db/db mice, demonstrated significant albuminuria at 14 weeks after birth compared to the control db/m mice which had maximum levels of albuminuria at 18 weeks (Fig. 8C). PDGF‐C levels were significant in diabetic db/db mice compared to the control mice at 10 weeks after birth. Urinary PDGF‐C levels peaked at 14 weeks and were significantly higher afterward compared to the control mice (Fig. 8D). Similar to the case of the STZ‐induced diabetic mice, plasma PDGF‐C levels in db/db mice were comparable to the control mice at 14 weeks (Fig. 8F). In conclusion, urinary PDGF‐C levels were elevated in diabetic mice in a similar fashion to that of albumin, an established early marker of diabetic kidney damage, indicating the possible adoption of urinary PDGF‐C as a prediction of kidney injury in diabetes.

## Discussion

In this study, we aimed to identify ChREBP target genes in mesangial cells and identify their roles on a genome‐wide scale by means of ChIP‐chip analyses for further understanding of the mechanism underlying diabetic glomerulopathy. Subtraction of ChREBP‐binding sites in mesangial cells cultured in a medium with normal glucose concentration, from that in a high glucose culture, revealed high glucose‐dependent binding of ChREBP to 139 sites across the genome, 15% of which are located in potential gene regulatory regions of 5 kb spanning 5′‐ or 3′‐UTR. Among them, the *PDGF‐C* gene was found to carry a cis‐aligned ChREBP‐binding site at approximately 1.5 kbp upstream of the transcriptional start site. Similarly, there was a ChREBP‐binding sequence in the first intron of the mouse *Pdgf‐c* gene as well. Quantitative PCR and immunoblot analyses revealed high glucose‐dependent upregulation of PDGF‐C expression in both human and mouse mesangial cells. ChIP analyses reproduced a direct binding of ChREBP to both human and mouse PDGF‐C genes in the presence of stimulation with high glucose, indicating an involvement of ChREBP in glucose‐mediated induction of PDGF‐C in mesangial cells in different species.

The PDGF system, comprising four isoforms (PDGF‐A, ‐B, ‐C, and ‐D) and two receptor chains (PDGFR‐*α* and ‐*β*), has been shown to play pivotal roles in wound healing, atherosclerosis, organ fibrosis, and cancers. Particularly, PDGF‐C is a potent mitogen for fibroblasts in vitro. A mouse model with transgenic overexpression of PDGF‐C in the heart demonstrated fibroblast proliferation, cardiac fibrosis, hypertrophies, and cardiomyopathy (Li et al. 2000; Ponten et al. 2003). Liver‐specific PDGF‐C overexpression by the transgene caused liver fibrosis and hepatocellular carcinoma (Campbell et al. 2005). A local PDGF‐C overexpression in the lung led to massive mesenchymal cell hyperplasia and death from respiratory insufficiency immediately after birth (Zhuo et al. 2006).

PDGF‐C expression in diabetic kidney disease has not been well documented. We found an increase in PDGF‐C expression in the glomerulus of STZ‐induced diabetic model mice by immunohistochemical methods. In the tubulointersitial area, we only detected basal expression of PDGF‐C in distal tubular epithelial cells, possibly as a constitutive expression in the normal kidney. Our coimmunofluorescent analyses indicated that the mesangial cell is a major site of PDGF‐C expression in the glomeruli in diabetes. Consistently, in cultured mesangial cells, PDGF‐C was induced by high glucose at mRNA levels. Knock‐down of the PDGF‐C gene in the mesangial cells abolished high glucose‐induced accumulation of type IV collagen and type VI collagen, suggesting an involvement of PDGF‐C in such fibrotic extracellular matrix production by the mesangial cells. In addition, stimulation of the mesangial cells with recombinant PDGF‐C promoted *COL4A1* and *COL6A1* mRNA expression within the cells. Moreover, mesangial cells express the cognate receptor PDGFR‐*α* (data not shown), and specific inhibition of the PDGF receptor by AG1295 diminished high glucose‐mediated induction of *COL4A1* and *COL6A1* mRNA, indicating a possible autocrine regulation of pathologic extracellular matrix expansion via PDGF‐C by mesangial cells in diabetic conditions.

On the other hand, PDGF‐C produced by glomerular mesangial cells may exert its influence via a paracrine mechanism. Indeed, analyses of various human kidney tubulointerstitial diseases typically revealed a finely granular immunohistochemical signal of PDGF‐C in the fibrosing area and the absence of intracellular localization of PDGF‐C in interstitial cells (Eitner et al. 2003), indicating a delivery of PDGF‐C from somewhere else. As such, not only tissue interstitial fluid but also glomerular filtrate might be candidates of the transit route for PDGF‐C, as we demonstrated elevation of urinary PDGF‐C in diabetic model mice. Interestingly, infusion of PDGF‐C accelerated glomerular capillary repair via PDGFR‐*α* on the glomerular endothelial cells in the early mesangiolytic phase of mesangioproliferative glomerulonephritis in rats (Boor et al. 2010). Therefore, this paracrine (or endocrine) mode of PDGF‐C action may constitute diverse roles in kidney disease, ranging from pathological to beneficial.

We also demonstrated that urinary PDGF‐C level is upregulated in the disease course of both type 1 and type 2 diabetic animal models. The elevation of PDGF‐C in the urine appeared at the same time or earlier than the elevation of albumin urinary excretion. Even though plasma PDGF‐C levels in diabetic animals were comparable to that in control mice, the origin of the elevated PDGF‐C in the urine is still elusive. There has been a recent effort to identify new biomarkers, ideally by noninvasive methods, which reflect kidney function, early injury, and/or repair that ultimately can relate to progression or regression of damage. The emerging biomarker candidates for DN include liver‐type fatty acid‐binding protein (Kamijo‐Ikemori et al. 2011), retinal venular caliber (Ikram et al. 2013), and urinary exosomal microRNAs (Barutta et al. 2013). As such, measurement of urinary PDGF‐C in diabetic patients is an area of great interest and studies are ongoing.

In conclusion, we propose a novel axis of ChREBP and PDGF‐C in extracellular matrix deposition, which might contribute to the development of glomerulopathy in diabetic conditions. Pharmacological inhibition or gene silencing in this axis seemed to ameliorate concomitant fibrotic gene regulation. To elucidate the value of targeting this cascade for therapeutic and diagnostic purposes, further analyses with clinical specimens are clearly needed.

## Conflict of Interest

No conflicts of interest, financial or otherwise, are declared by the authors.

## Supporting information




**Table S1.** The peak location of ChERBP binding site and the nearest gene list.
